# Resistance to and Accumulation of Heavy Metals by Actinobacteria Isolated from Abandoned Mining Areas

**DOI:** 10.1155/2015/761834

**Published:** 2015-02-11

**Authors:** Soraia El Baz, Mohamed Baz, Mustapha Barakate, Lahcen Hassani, Abdelhay El Gharmali, Boujamâa Imziln

**Affiliations:** ^1^Environmental Microbiology and Toxicology Unit, Laboratory of Biology and Biotechnology of Microorganisms (LBBM), Faculty of Sciences Semlalia, Cadi Ayyad University, P.O. Box 2390, 40000 Marrakech, Morocco; ^2^Laboratory of Hydrobiology, Ecotoxicology and Assainissement (LHEA), Faculty of Sciences Semlalia, Cadi Ayyad University, P.O. Box 2390, 40000 Marrakech, Morocco

## Abstract

Accumulation of high concentrations of heavy metals in environments can cause many human health risks and serious ecological problems. Nowadays, bioremediation using microorganisms is receiving much attention due to their good performance. The aim of this work is to investigate heavy metals resistance and bioaccumulation potential of actinobacteria strains isolated from some abandoned mining areas. Analysis of mining residues revealed that high concentration of zinc “Zn” was recorded in Sidi Bouatman, Arbar, and Bir Nhass mining residues. The highest concentration of lead “Pb” was found in Sidi Bouatman. Copper “Cu,” cadmium “Cd,” and chromium “Cr” were found with moderate and low concentrations. The resistance of 59 isolated actinobacteria to the five heavy metals was also determined. Using molecular identification 16S rRNA, these 27 isolates were found to belong to *Streptomyces* and *Amycolatopsis* genera. The results showed different levels of heavy metal resistance; the minimum inhibitory concentration (MIC) recorded was 0.55 for Pb, 0.15 for Cr, and 0.10 mg·mL^−1^ for both Zn and Cu. Chemical precipitation assay of heavy metals using hydrogen sulfide technic (H2S) revealed that only 27 isolates have a strong ability to accumulate Pb (up to 600 mg of Pb per g of biomass for *Streptomyces* sp. BN3).

## 1. Introduction

Heavy metal pollution is one the most important environmental problems in marine, terrestrial, and freshwater areas [1]. They can be accumulated in the human body through food webs, thereby promoting chronic and acute disorder and creating obviously serious health problems [2, 3]. The anthropogenic sources of metal contamination can be divided into five main groups: (i) metalliferous mining and smelting, (ii) industries, (iii) atmospheric deposition, (iv) agriculture, and (v) waste disposal [4, 5]. Industries bearing heavy metals, such as Pb, Zn, Cr, Cd, Cu, As, and Ni, are a major concern because they are well known to produce a long-term negative impact on soil and water in the surrounding environment [6, 7].

Conventional technologies for removing metals from industrial effluents, such as chemical precipitation, chemical oxidation or reduction, ion exchange, membrane filtration, electrochemical treatment, reverse osmosis, and evaporation, were frequently used. These processes may be ineffective or extremely expensive [8]. Nowadays, new processes, based on microorganism technology like bioaccumulation and biosorption, have been investigated for the developments of cheaper and more effective technologies in order to decrease the amount of industrial wastewater produced and to improve the quality of the treated effluent. A wide variety of microorganisms, for example, bacteria, yeast, algae, protozoa, and fungi, have developed the capabilities to protect themselves from heavy metal toxicity by various mechanisms such as uptake, oxidation, adsorption, methylation, and reduction [4]. It was also reported that some bacteria can use mechanisms of tolerance and detoxification of heavy metals and still produce chelating agents that bound metals and reduce their toxicity [9]. Many living bacteria have been reported to reduce or to transform toxic contaminants into their less toxic forms. Actinobacteria constitute a morphologically diverse group of Gram-positive bacteria, with high metabolic versatility [10]. As reported earlier, actinomycetes are the good bioremediation [11–16]. Their metabolic diversity, particular growth characteristics, mycelial form, and relatively rapid ability to colonize selective substrates make them well-suited for use as agents for bioremediation of inorganic and organic compounds [16–20].

Industrial and mining activities are important for economic development, especially in many developing countries as Morocco. However, these activities also represent the main sources of heavy metal contamination.

In Marrakech region, many exploitation metal mines have ceased to be functional many years ago. Mining residues were abandoned in open air without any treatment and were exposed to the atmospheric hazards. In fact, these release heavy metals and other pollutants to the drainage and represent a potential danger of surrounding terrestrial and aquatic ecosystems. Moreover these mining sites are generally situated near agricultural zones [21].

To our knowledge, little research had been done regarding the problematic of mining residues in our country, despite awareness of health and environmental risks that they may cause.

**Figure 1:**
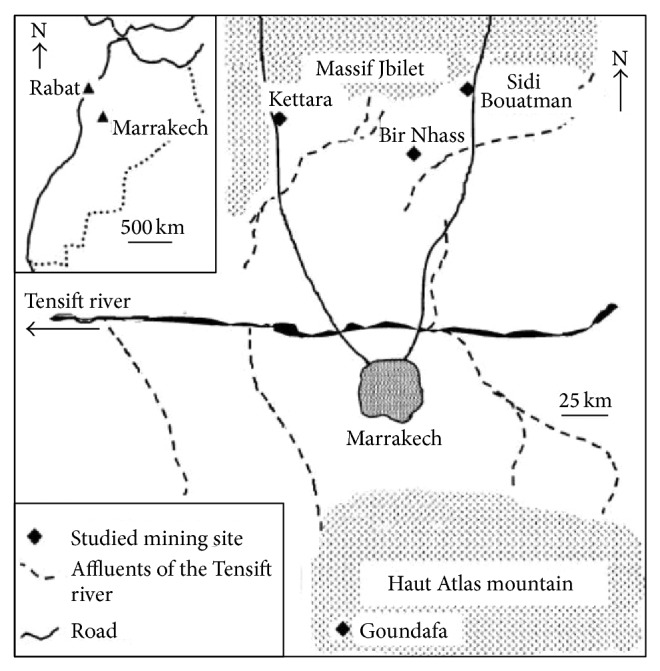
Localisation of prospected abandoned mining areas of Marrakech, Morocco: Kettara, Sidi bouatman, Bir Nhass, and Goundafa.

In the present study we are interested in mining residues collected from various abandoned mining areas located around Marrakech region, West Central Morocco (Figure 1). Kettara mine (KT) is located approximately 30 km North-West of Marrakech and was operated for pyrrhotine extraction until 1981 [22]. Sidi Bouatman mine (SB) is located approximately 33 km north of Marrakech and was operated for Pb, Zn, and pyrite extraction until 1980. The old Bir Nhass zinc mine (BN) is located approximately 30 km north of Marrakech; it was closed at the end of the 20th century. Goundafa mine, located approximately at 90 km south of Marrakech, operated for Pb, Zn, and Cu extraction and in 1989 the mine has ceased to be functional. In Goundafa area, samples were collected from two sites Arbar plant (GA) and Tenfit Mine (GT).

The aim of the present work deals with the evaluation of the contamination levels of mining residues, the isolation of heavy metals resistant actinobacteria, their potential capacity to accumulate heavy metals (Pb, Cu, Zn, Cd, and Cr), and both morphological and molecular characterization of resistant and accumulating isolated strains.

## 2. Materials and Methods

### 2.1. Total Concentration of Heavy Metals of Mining Residues Samples

The pH and the electrical conductivity (EC) for each mining residue sample were measured according to AFNOR X31-103 [23] and [24], respectively. The total concentrations of Zn, Pb, Cd, Cu, and Cr were determined for all sample residues [23]. Samples were oven-dried at 500°C, finely ground (2 mm), and then ashed in a crucible. 5 mL of fluohydric acid (50%) was added to 0.5 g of each sample and brought to dry. Samples were then digested with* aqua regia* (HCl and HNO_3_, successively, in a ratio of 3 : 1). Flasks, containing samples, were heated gently until the samples were digested, which is indicated by the formation of a clear solution above the residue. This solution was adjusted to 10 mL with distilled water. Digested mining residues samples were analyzed for metals concentrations (Pb^2+^, Cu^2+^, Zn^2+^, Cr^6+^, and Cd^2+^) using an UNICAM 929 model flame atomic absorption spectrophotometer (FAAS).

### 2.2. Isolation of Actinobacteria from Mining Residues

Ten grams of the residues sample were suspended in 100 mL of sterile distilled water and shaken vigorously for 10 min. Aliquots (0.1 mL) of appropriate dilutions were plated on Bennett's agar medium [25]. Plates were incubated at 30°C for 15 days. The isolation and purification of actinobacteria were done by repeated streaking and dilution-plate techniques using Bennett's agar medium. All isolates were then maintained in glycerol 20% at −20°C.

### 2.3. Selective Medium for the Metal-Resistant Actinobacteria

In order to choose the appropriate medium to select the metal resistant actinobacteria, two media were used, nutrient agar (type Pronadisa), and modified Duxbury agar (KCl: 0.3 g·l^−1^; CaCl_2_: 0.025 g·l^−1^; MgSO_4_·7H_2_O: 0.2 g·l^−1^; (NH_4_)_2_SO_4_: 0.5 g·l^−1^; glucose: 1 g·l^−1^; tryptone: 1 g·l^−1^; yeast extract: 0.5 g·l^−1^; agar: 15 g·l^−1^), the pH of the medium was adjusted to 7.00 [26]. Tested metals were: lead [Pb(NO_3_)_2_]; copper [Cu(NO_3_)_2_·H_2_O]; cadmium [Cd(NO_3_)_2_·4H_2_O]; chromium [K_2_Cr_2_O_7_] and zinc [Zn(NO_3_)_2_·6H_2_O]. Stock solutions of metals salts, prepared in distilled water were sterilized by filtration (0.20 *μ*m). 0.05 mg·mL^−1^ of each heavy metal was used in both media (nutrient agar and Duxbury agar). Plates were incubated at 30°C for 7 days. Growth of actinobacteria on culture media containing no metals was used as control.

### 2.4. Screening of Heavy Metal Resistant Actinobacteria

Heavy metal resistance of all actinobacteria isolated from mining residues was assessed against five elements (Pb, Cu, Zn, Cd, and Cr) in triplicate with different concentrations (0.05, 0.15, 0.25, and 1 mg·mL^−1^) in Duxbury agar. Growth percentages were calculated for each metal after comparison with controls.

To determine the MIC of each metal, actinobacteria isolates were grown in Duxbury agar with increased concentrations. The MIC was defined as the lowest concentration of the metal inhibiting the visible growth of the bacteria.

### 2.5. Screening of Heavy Metal-Accumulating Actinobacteria

Actinobacterial isolates were streaked on Duxbury agar plates containing 0.05 mg·mL^−1^ of each tested metal. After incubation, plates were exposed to hydrogen sulfide gas in a sealed container (resulting from the reaction of Na_2_S with HCl). Simple and effective formation of dark precipitate was achieved for several metals by treatment with sulfide. Upon formation of the metal sulfide, plates were carefully screened to detect any change in the region surrounding bacteria colonies [27].

In order to determine metal quantities accumulated by isolates, actinobacteria strains were cultured on Bennett liquid medium without heavy metals. After incubation (30°C for 7 days), cultures were centrifuged at 12,000 rpm for 20 min; the pellets were washed twice with phosphate-buffered saline and were resuspended in sterile solutions containing 0.5 mg·mL^−1^ of tested metal. The tubes were then incubated at room temperature in a roller mixer. After 3 h of incubation, the cells were harvested by centrifugation at 12,000 rpm for 20 min. The amount of residual metal present in the supernatant was measured by an atomic absorption spectrophotometer. At the same time, dry weight of the bacterial pellet was determined. Values are expressed as the averages of three determinations carried out in parallel. Bioaccumulation of the metal in the biomass was expressed as the amount removed from solution containing the tested metal based on the following equation [3]:

(1) $$\begin{array}{c} \text{Metal}\;\text{removal}=\frac{\left[C_{0}-C_{t}\right]V}{M}, \end{array}$$

where *C*_0_ is metal initial concentration in the solution (mg·mL^−1^); *C*_*t*_ is metal concentration after 3 h in the solution (mg·mL^−1^); *V* is total volume of the culture, and *M* is dry mass of biomass (g).

### 2.6. Morphological and Physiological Characterization of Actinobacteria Isolates

The morphological, cultural, physiological, and biochemical characteristics of the selected isolates were evaluated as described by Shirling and Gottlieb [28]. Cultural characteristics were observed on glycerol-asparagine agar (ISP5), inorganic salts-starch agar (ISP4), tryptone-yeast extract agar (ISP1), and yeast extract-malt extract agar (ISP2) media at 30°C for 7–21 days. Melanin production was detected by growing the isolates on peptone-yeast extract-iron agar (ISP6) [28]. The substrate mycelium color was identified in terms of Prauser's guide 7 [29]. The assimilation of carbohydrates was studied by using the medium ISP9, containing 13 different carbohydrates at a concentration of 1% (w/v) as sole carbon source.

### 2.7. Molecular Identification of Actinobacteria Isolates

The DNA of selected actinobacteria was isolated according to the procedure described by Hopwood et al. [30]. The 16S rDNA was amplified using the PCR method with Taq DNA polymerase and primers 27F (5′-AGAGTTTGATCMTGGCTCAG-3′) and 1492R (5′-TACGGYTACCTTGTTACGACTT-3′) [31]. The conditions for thermal cycling were as follows: denaturation of the target DNA at 98°C for 3 min followed by 30 cycles at 94°C for 1 min, primer annealing at 52°C for 1 min, and primer extension at 72°C for 5 min. At the end of the cycling, the reaction mixture was held at 72°C for 5 min and then cooled to 4°C. The PCR product obtained was sequenced using an automated sequencer and the same primers as above for sequence determination (Macrogen Inc. (Seoul, Korea)). The sequence was compared for similarity with the reference species of bacteria contained in genomic database banks, using the NCBI Blast available at http://www.ncbi.nlm.nih.gov/. A phylogenetic tree based on 16S rRNA gene sequence was constructed with the neighbor-joining algorithm in MEGA version 5 [32].

### 2.8. Siderophore Assays

The universal chemical assay of Schwyn and Neilands [33] was used for detecting siderophore production by actinobacteria using Chrome Azurol S agar assay (CAS). For this experiment, glasswares were first cleaned by immersion in dichromate-acid solution for 48 h and then rinsed several times with distilled water before use. In parallel, a minimum medium agar (MM) without iron (glucose: 10 g·l^−1^; KNO_3_: 1 g·l^−1^; MgSO_4_: 0.5 g·l^−1^; KCl: 0.5 g·l^−1^; K_2_HPO_4_: 0.5 g·l^−1^; agar: 15 g·l^−1^) was prepared. Pure strains of actinobacteria were grown on MM for 3 days at 30°C and then mycelia plugs (9 mm in diameter) were cut, placed on CAS agar plates, and incubated for 3 days at 30°C. As control, MM was amended with 0.01 g·l^−1^ of FeSO_4_·7H_2_O. A positive reaction is indicated by a color change of CAS reagent from blue to orange, leading to a clearly visible yellow-orange halo around the disk.

Hydroxamate-specific assay was used to reveal the presence of hydroxamate siderophores in culture supernatants. Siderophore production was monitored by ferric-catalyzed acid hydrolysis and Csaky assay [34]. Strains producing siderophores were inoculated on liquid minimum medium MM. The cultures were incubated for 2 weeks and then centrifuged at 4000 rpm for 20 min at 4°C. the supernatant (2 mL) was put in a test tube. This sample was saturated with ferric ion, and a 0.2 mL aliquot was hydrolyzed with 2 mL of 3 M sulfuric acid for 4 h in a 120°C (autoclave). Sodium acetate (2 M; 7 mL) and sulfanilamide (2 mL of a 1% [w/v] solution in 30% [v/v] acetic acid) were added, and the pH was adjusted to 3.1 ± 0.1. This sample was oxidized with iodine (2 mL of 0.0325% [w/v] I_2_ in 0.05% [w/v] KI) for 4 min at room temperature (24°C). Excess iodine was removed by the addition of sodium arsenite (2 mL of a 0.075% [w/v] solution in distilled water). N-(1-Naphthyl)ethylenediamine (2 mL of a 0.05% [w/v] solution in distilled water) was added, and the mixture was allowed to stand for 30 min to complete the color development. The solution was diluted to 50 mL, and the* A*_543_ was measured. A blank was prepared by addition of the reagents to 0.2 mL of distilled water.

The presence of catechol siderophores was also investigated in culture supernatants using a modified version of the Arnow reaction [35, 36]. In this assay, the test sample is treated with HCl, NaNO_2_, and Na_2_MoO_4_-2H_2_O, which results in a yellow color; subsequent addition of NaOH causes the color to change to red in the presence of catechol compounds. Reagents were prepared and reactions carried out as in Neilands and Nakamura [36]; absorbance values were determined using a spectrophotometer at a wavelength of 515 nm.

### 2.9. Statistical Analysis

All experiments were performed in triplicate. To compare differences in metal accumulation among tested isolates, obtained data were analyzed through multivariate analysis of variance (ANOVA), and the means were compared by Tukey multiple range test. All numeric differences in the data were considered significantly different at the probability level of *P* ≤ 0.05.

## 3. Results

### 3.1. Assessment of Present Mining Residues Heavy Metal Concentrations

The mean metal concentration (in g·Kg^−1^) in all prospected mining residues and their related statistical parameters is shown in Table 1. Heavy metal concentrations did show variation between sites. At Sidi Bouatman site, Pb and Zn were the dominant elements with 10.19 and 18.87 g·Kg^−1^, respectively. While At Arbar plant, the dominant element was Zn with 14.43 g·Kg^−1^. In Kettara mine, Cu was found to be the dominant metal with 2.94 g·Kg^−1^; for Bir Nhass, Zn with 9.99 g·Kg^−1^, and at Tenfit mine, the higher concentration of the dominant metal (Zn) reached 2.09 g·Kg^−1^. Mean concentrations of Cr and Cd were significantly lower than the other tested metals in all sites. For the pH and conductivity values, there were no significant variations between the four sites (Bir Nhass, Sidi Bouatman, Arbar plant, and Tenfit mine), while a significant decrease in pH values (2.2) and a significant increase in conductivity (10.28 mS·cm^−1^) were recorded in Kettara site.

### 3.2. Isolation of Actinobacteria and Screening of Metal-Resistant Strains

A total of 59 actinobacteria strains were isolated from mining residues located in different mining areas around Marrakech city (BN, 30 isolates; SB, 8 isolates; and GT, 21 isolates). No actinobacteria strain was isolated from Kettara and Arbar sites.

**Figure 2:**
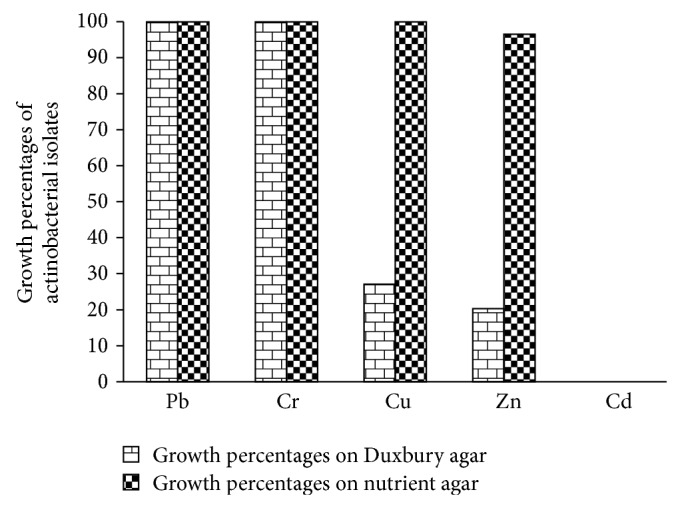
Growth percentages of actinobacterial isolates on both Duxbury agar and nutrient agar with: Pb, Cu, Zn, Cd, and Cr at the concentration 0.05 mg·mL^−1^ (the percentage of growth was calculated compared to the control).

Bacterial resistance to heavy metals was performed in two culture media (nutrient agar and modified Duxbury agar). Obtained results shown in Figure 2 indicate differences in growth of strains, in both media amended or not with heavy metals (Cd, Cu, Pb, Zn, and Cr). In the presence of metals, growth of tested isolates on Duxbury agar was very low in comparison with nutrient agar. On Duxbury agar supplemented with metals, the growth percentages reached 100% for Pb, and Cr, while the growth percentage did not exceed 28% for Cu and 21% for Zn. On nutrient agar, these percentages were found to be equal to 100% for Pb, Cu, and Cr and 96.61% for Zn. From these results, it seems clear that Duxbury agar did not affect or affects slightly the biodisponibility of metal in the culture medium. For this reason, we have chosen Duxbury medium as a suitable medium to select heavy metals resistant actinobacteria.

Resistance of 59 actinobacteria isolates to the five heavy metals (Pb, Cu, Cd, Zn, and Cr) was run, and MIC values were listed in Table 2. According to the obtained data, maximal MIC values were 0.55 mg·mL^−1^ for Pb, 0.15 mg·mL^−1^ for Cr and 0.1 mg·mL^−1^ for Zn, and Cu for Cd; no strain was found to be resistant.

From data in Table 3, differences in heavy metal toxicity levels against the tested strains were reported. One hundred percent of the tested strains were capable to grow in the presence of Pb and Cr at 0.05 mg·mL^−1^. However, in the presence of Cu and Zn at the same concentration, only 30.08 and 34.05% of actinobacteria strains were able to grow, respectively. For lead, the percentage of resistant bacteria decreased with the increase of its concentration in the medium. When concentration of Pb reached 0.25 mg·mL^−1^, the percentage of resistant strains decreased to 55.40%, and the growth was completely stopped at 1 mg·mL^−1^. For Copper, Zinc, and Chromium, actinobacterial growth was completely repressed for concentrations greater or equal to 0.15 mg·mL^−1^.

From these results, lead may be the most tolerated metal by our selected strains, and cadmium was the most toxic one. Chromium, zinc, and copper gave intermediate results. In this report, we have also noted that resistance percentages to Pb, Zn and Cr among strains isolated from Sidi Bouatman site (Tables 2 and 3), were higher compared to isolates from other sites. We can conclude that the resistance levels depended on the site of strains isolation.

### 3.3. Screening of Heavy Metal-Accumulating Actinobacteria

**Figure 3:**
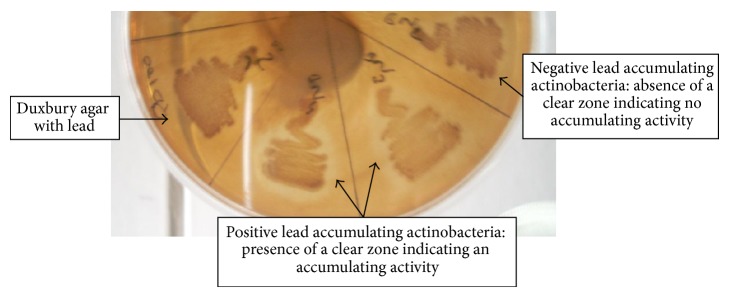
Example of positive and negative lead accumulating actinobacteria on Duxbury agar supplemented with Pb.

According to the finding results, 27 of the total isolates were found to be able to accumulate lead on lead-Duxbury agar. We notice the presence of a clear halo around the colony (Figure 3), which shows the disappearance of metal and this may be due to its accumulation by the strain. But none of strains was seen to play some accumulation capacities against Cu, Cd, Cr, or Zn.

**Figure 4:**
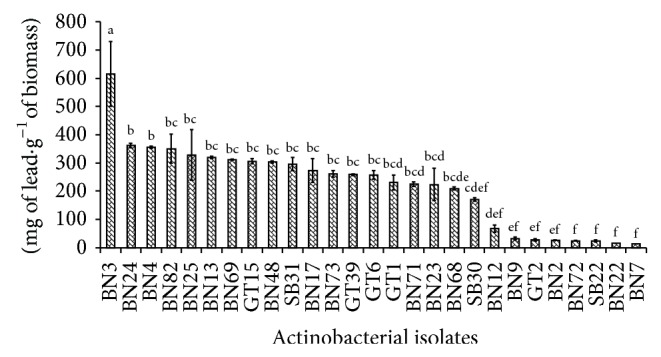
Quantities of removed lead by the tested actinobacteria strains.

Accumulated quantity results of lead by actinobacterial tested strains are shown in Figure 4. Among the twenty seven tested strains, the amount of accumulated lead ranged from 12.98 mg·g^−1^ (BN7) to 615.04 mg·g^−1^ (BN3). According to statistical analysis, there was a significant difference (*P* ≤ 0.05) between the accumulation capacity results, and this difference may depend on tested strain.

### 3.4. Taxonomic and Molecular Identification of the Actinobacterial Isolates

Selected isolates (27 strains) were tested for taxonomical diversity using morphological, cultural, physiological, and biochemical criteria as well as other features (Table 4). The aerial and substrate mycelium colors were determined on ISP2, ISP4, and ISP5 media. Tested strains showed different abilities to assimilate 13 carbon sources (Table 4). The analysis of 16S rRNA gene sequence of these strains (Table 5 and Figure 6) confirmed this classification. Phylogenetic tree, based on 16S rRNA gene, clearly demonstrated that the majority of selected isolates belong to* Streptomyces* genus and only three isolates (GT6, GT15, and GT39) were found belonging to* Amycolatopsis* genus.

The 16S rRNA sequence was deposited in GenBank under accession numbers: KF479164, KF479165, KF479166, KF479167, KF479168, KF479169, KF479170, KF479171, KF479172, KF479173, KF479174, KF479175, KF479176, KF479177, KF479178, KF479179, KF479180, KF479181, KF479182, KF479183, KF479184, KF479185, KF479186, KF479187, KF479188, KF479189, and KF479190, respectively, for* Streptomyces* strains BN2, BN3, BN4, BN7, BN9, BN12, BN13, BN17, BN22, BN23, BN24, BN25, BN48, BN68, BN69, BN71, BN72, BN73, BN82, GT1, GT2, GT6, GT15, GT39, SB22, SB30, and SB31.

### 3.5. Siderophore Analysis

**Figure 5:**
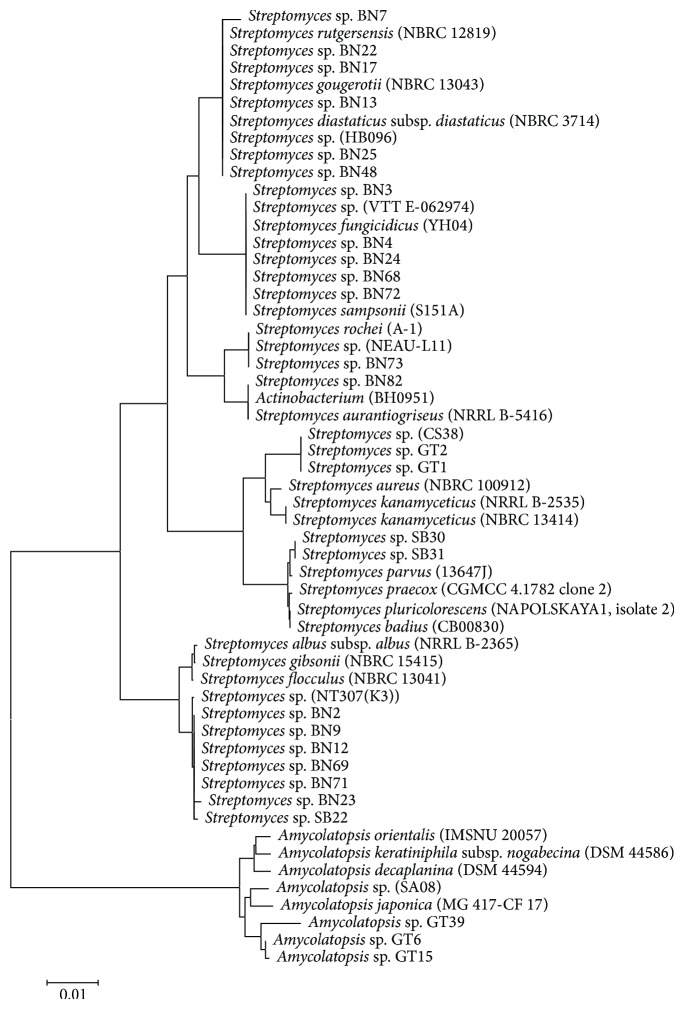
Evolutionary relationships of taxa: the evolutionary history was inferred using the neighbor-joining method [71]. The optimal tree with the sum of branch length = 0.21501137 is shown. The tree is drawn to scale, with branch lengths in the same units as those of the evolutionary distances used to infer the phylogenetic tree. The evolutionary distances were computed using the maximum composite likelihood method [72] and are in the units of the number of base substitutions per site. The analysis involved 55 nucleotide sequences. Codon positions included were 1st + 2nd + 3rd + noncoding. All positions containing gaps and missing data were eliminated. There were a total of 1392 positions in the final dataset. Evolutionary analyses were conducted in MEGA5 [32]. The scale bar represents 0.01 substitutions per nucleotide position.

**Figure 6:**
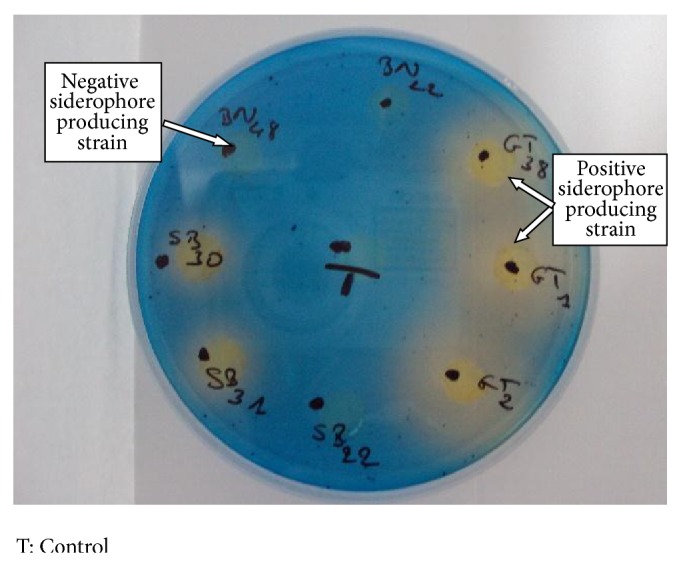
Screening of siderophore producing actinobacteria using chrome azurol S agar (positive strain are yellow-orange, negative strain are blue).

For this parameter, 27 lead accumulating actinobacterial isolates were checked for siderophore secretion. Strains were cultivated on CAS medium for 7 days and, when a red or orange halo appeared around colonies, strain was considered as positive for the secretion of siderophore and was able to chelate iron from the culture medium (Figure 5). The universal siderophore assay was positive for only 12 of the 27 tested isolates (BN4, BN17, BN24, BN68, BN72, BN82, GT1, GT2, GT6, GT15, SB30, and SB31).

All the 12 siderophore positive strains were able to produce hydroxamate siderophore type, and none of them was able to produce catecholate siderophore.

## 4. Discussion

The aim of this study was to select actinobacteria strains from heavy metal contaminated sources of Moroccan abandoned mines. Heavy metal resistance and accumulation capacities of selected strains were performed in order to investigate their applicability as a bioremediators for polluted areas.

Physicochemical analysis of mining residue samples revealed the presence of high concentrations of heavy metals in all studied sites especially for Pb, Cu, and Zn. In general, these results were in accordance with previous studies carried out in abandoned mines in Morocco [21] and in others countries such as Sweden, Canada, and Spain [37–40]. Excepting Kettara residues samples, all samples presented a neutral to slightly alkaline pH explained by the presence of high concentration of carbonates. Furthermore, the reason behind acidic pH in Kettara mine is related to the oxidation of sulfuric acid which, in the absence of sufficient neutralizing components, brought down the pH [41–43]. These proprieties may affect directly living bacteria's diversity and ecophysiology in the prospected sites. In the present study, the acidic pH may explain why we did not isolate any actinobacteria strain from the Kettara site. Moreover, our results showed a negative correlation between actinobacterial diversity and heavy metal pollution levels. Indeed, we have isolated more actinobacterial strains in BN (30 isolates) and GT (21 isolates) than in SB site (08 isolates). Several authors pointed out that heavy metal pollution of aquatic and soil ecosystems induce a decrease in the microbial diversity [5, 9]. Haferburg and Kothe [4] reported that there was a clear mutual influence in mining areas: not only are microbes in soil affected by their environment directly and indirectly but also they control particular soil parameters. These authors suggested also that growth and metabolism can lead to changes in pH, redox potential, and ionic strength of the soil.

From the prospected mining sites, we have isolated a total of 59 actinobacteria strains. All isolates were screened for their resistance to Pb, Zn, Cd, Cu, and Cr. In order to assess the applicability and the suitability of culture medium in screening heavy metal resistant bacteria, we have used two culture media (rich medium, nutrient agar and minimum medium, Duxbury agar).

Obtained data revealed that all isolates streaked on nutrient agar with metals did not show any significant difference in growth comparing to the control one. In contrast, a significant difference in growth was observed for strains tested on Duxbury agar with metals in comparison with the control. We suggest that this is not only due to the metal kind but also due to the culture medium composition. Indeed, the medium composition may modulate the metal availability and consequently its toxicity. Nutrient agar containing organic compounds can chelate metals and make it not available for the tested microorganisms [16, 44]. In contrast, when isolates were streaked on Duxbury agar with metals, only some of them showed resistance ability. So, the latter medium may be considered as a more suitable support for such studies. Our findings agree with other reports in which frequently minimal medium has been used. Indeed, complex media are inadequate due to the high concentration of organic components that absorb metallic ions. Moreover, some heavy metals have high affinity to bind organic matter implying a decrease of their bioavailability [16, 44]. The same observations were reported by Majzlik et al. [45]. Schmidt et al. [46] reported that a* Streptomyces* strain grew very differently depending on growth media (minimal medium and TSB complex medium) amended with nickel.

Various methods of actinobacteria classification are used elsewhere [47]. In several works, the combination of morphological and chemical properties with 16S rRNA sequencing has been used to differentiate between actinobacteria genera [48]. The 16S rRNA gene sequencing of our selected isolates demonstrated that 88.9% of these organisms are phylogenetically related to members of the* Streptomyces* genus, and 11.1% are closely related to members of the* Amycolatopsis* genus. Although, the neighbor-joining method showed that some isolates belong to the same species, they have different physiological and biochemical features. Further analyses (as DNA-DNA hybridization) are required to clarify the systematic belonging of some of our selected strains.

It is generally assumed that environmental heavy metal pollution leads to the establishment of a tolerant or resistant microbial population [49]. In the present study, some of the tested strains could resist relatively high concentrations of lead (up to 0.55 mg·mL^−1^ for* Streptomyces *sp. BN2) and hexavalent chromium (up to 0.10 mg·mL^−1^). Polti et al. [16] have previously isolated nine chromium resistant actinobacteria strains from contaminated sites in Tucumán (Argentina), which were identified as members of the genus* Streptomyces*. Resistance of actinobacteria to Pb was previously reported by Sanjenbam et al. [50] who have described a maximum tolerance concentration of* Streptomyces* that reaches 1.8 mg·mL^−1^.

Our isolates could also resist Cu and Zn (0.10 mg·mL^−1^) and corroborate those of Albarracín et al. [51] who showed that* Amycolatopsis tucumanensis* strains, isolated from copper-polluted sediments, were resistant to concentrations up to 0.08 mg·mL^−1^ of CuSO_4_. In the other hand, none of our isolates were able to grow in the presence of Cadmium. A similar observations were recorded for* Streptomyces coelicolor* A3(2) isolated from an abandoned uranium mining area [46].* Amycolatopsis *sp. GT6,* Amycolatopsis *sp. GT15, and* Amycolatopsis* sp. GT39 can tolerate very high concentrations of the four metals tested (Pb, 0.25; Cu, 0.10; Cr, 0.15; and Zn, 0.10 mg·mL^−1^). From obtained results, it appeared that actinobacterial MICs values which are determined with traditional media did not agree with metal concentrations detected in sites. Selected strains are unable to resist to heavy metal's concentrations equal to those recorded in mining residue samples. This may be explained by the presence of nonavailable forms of metals in sites with high percentages. In a previous study on the same sites (Sidi Bouatman, Bir Nhass, and Goundafa) heavy metals (Pb, Cu, Cd and Zn) are generally unavailable (Table 6) [21]. Moreover, some interactions can alter chemical speciation and the bioavailability of these micropollutants. In the same way, some authors have pointed out that the soluble and exchangeable fractions of heavy metals largely determine the availability and mobility of heavy metals [52, 53]. Furthermore, pH is one of the most decisive factors who act on the solubility of heavy metals. In addition, the presence of limestone in Goundafa and Sidi Bouatman residues may explain the low availability of their toxic form of heavy metals. According to these observations we can conclude that our selected isolates had abilities to tolerate relative high metal concentrations than what is available in sampling sites. This may also explain why all isolates were sensitive to cadmium.

Metal accumulation or biotransformation is alternative mechanisms for metal detoxification used by bacteria [54]. Chemical precipitation taste of heavy metals using hydrogen sulfide technic (H_2_S) revealed that only 27 isolates have a strong ability to accumulate Pb; the accumulated concentrations recorded high levels (up to 600 mg of Pb per g of biomass for* Streptomyces* sp. BN3). As it had been already pointed out by Hernández et al. [27], the technic used to detect heavy metal accumulator actinobacterial strains did give satisfactory results. Obtained results show that selected isolates are able to accumulate only lead metal. Similarly, Rho and Kim [55] reported the capacity of* Streptomyces viridochromogenes* to accumulate lead up to 164 *μ*g·mg^−1^. Significantly, lead adsorption was much higher for various fungi including* Mucor rouxii* (769 mg·g^−1^) [56] and* Paecilomyces marquandii* (324 mg·g^−1^) [57].* Streptomyces albus* was also able to adsorb copper and gold as well as lead in its cell wall [58]. In contrast,* A. tucumanensis* showed the highest bioaccumulation value for growing cells (25 mg·g^−1^) among other microorganisms previously proposed for bioremediation processes [51].* S. zinciresistens* accumulated Zn^2+^ (26.81 mg·g^−1^) and Cd^2+^ (85 mg·g^−1^) mainly on the cell wall followed by intracellular accumulation [59]. The surface envelopes of bacterial cells can adsorb various heavy metals by means of ionic bonds to their intrinsic chemical groups [55]. Anionic groups such as carboxylate and phosphate groups of peptidoglycan and teichoic acids are considered as the major metal binding sites [55].

The secondary metabolites produced by actinobacteria may enable the bacteria to cope with stress factors including toxic levels of heavy metals [14]. Another study reveals that a* Streptomyces* strain, producing a siderophore, was capable of sequestering a range of ions including Mn, Co, Cd, Ni, Al, Li, Cu, Zn, and Mg [60]. In the present study, among 27 Pb resistant and accumulating strains, only 12 were found to be able to produce siderophore from hydroxamate family. These findings suggest that there is no relationship between metal accumulation capacities and production of siderophore especially in iron rich environs. The exploitation of these isolates in bioremediation of metal contaminated iron deficient soils may be useful tool. In the same way, some authors suggested application of these kinds of bacteria as an interface with plants able to accumulate metals [61, 62].

In the other hand, we have recorded that* Streptomyces* sp. BN2 strain shows a high resistance level to Pb (0.55 mg·mL^−1^) and a low accumulation capacity of this metal (26.37 mg·g^−1^).* Streptomyces* sp. BN3 strain, which is twofold less resistant to Pb than* Streptomyces* sp. BN2 strain, was able to accumulate more than 26 times the quantity of Pb than* Streptomyces* sp. BN2.* Streptomyces* sp. BN48 strain showed a low resistance level (0.20 mg·mL^−1^) and presented an intermediate accumulation capacity (282.74 mg·g^−1^) in comparison with* Streptomyces* sp. BN3 and* Streptomyces* sp. BN2. Consequently, it can be clearly noticed that there was no correlation between resistance level and accumulation capacity for tested strains.

In fact, pollution levels may influence the genetic make-up of resident microorganisms. Members of* Streptomyces* and* Amycolatopsis* genera can survive in polluted environments with heavy metals as mentioned by Polti et al. [16] and Albarracín et al. [17]. Álvarez et al. [63] reported that the presence of heavy metal resistant strains in different* Streptomyces* clades may have two different explanations: (i) the resistance was already present in the most recent common ancestor (MRCA) and was then inherited by the different lineages; (ii) the different lineages inherited from the MRCA the capacity to develop new mechanisms or modify existing ones, in order to generate resistance to heavy metals. Schütze and Kothe [64] indicated that several morphological, physiological, and reproductive characteristics of* Streptomyces* (the filamentous growth, the formation of hyphae, and the production of spores) would allow its species to occupy extreme environments. Since these properties are shared by all species of the genus, this can be interpreted as evidence that supports the inheritance of resistance to heavy metals from the MRCA; and this is in agreement with the work done by Zhou et al. [65].

Many processes could be used in the bioremediation of contaminated wastewaters and soils. Microorganisms as actinobacteria might be a good tool which can be used in a bioreactor configuration to treat metal contaminated wastes prior to discharge into the environment. Recently, reports pointed out that such microorganisms could be used in large-scale biofilm bioreactors for the treatment of metal-laden wastewater [66–70]. In the present study, living cells of actinobacteria gave satisfactory metal accumulating results especially for lead. More studies should be designed at molecular levels to evaluate and to identify mechanisms involved in the association between metal resistances and accumulation in order to upgrade the bioremediation potential of theses strains. These studies seem to be highly promising in terms of the creation of fields that encourage the development of bioremediation processes using native microorganisms.

## 5. Conclusion

Mining sites are considered suitable for the isolation of heavy metal resistant microorganisms and useful sources of potential bioremediators. Many abandoned mining sites are highly contaminated with metallic elements. It is very interesting to restore them especially by combining several environment friendly remediation techniques and by improving their quality. In our opinion, some of our selected actinobacteria can play very significant roles in the remediation of contaminated sites. However, further studies are required to enhance bioremediation potentialities of our strains to perform biological process and to optimize their role in removing heavy metals from contaminated environments.

## Figures and Tables

**Table 1:** Mean heavy metal concentrations, pH, and conductivity in mining residues samples.

Mining areas	Mining residues content						
	Heavy metals content g·Kg^−1^					pH	Conductivity (mS·cm^−1^)
	Pb	Cu	Zn	Cd	Cr		
Kettara	0.028 ± 0.007	2.942 ± 0.134	0.685 ± 0.092	ND	0.017 ± 0.002	2.20	10.28
Bir Nhass	1.407 ± 0.341	0.095 ± 0.024	9.989 ± 1.223	0.075 ± 0.011	0.031 ± 0.005	6.52	2.21
Sidi Bouatman	10.189 ± 3.580	0.102 ± 0.029	18.870 ± 5.733	0.044 ± 0.017	0.035 ± 0.005	7.42	2.08
Arbar plant	1.619 ± 0.432	1.243 ± 0.079	14.434 ± 0.928	0.070 ± 0.007	0.006 ± 0.001	7.17	2.27
Tenfit mine	0.098 ± 0.002	0.498 ± 0.068	2.093 ± 1.020	ND	0.014 ± 0.001	7.52	1.46

^*^ND: not detected.

**Table 2:** Heavy metals tolerance in actinobacteria from mining areas.

Mining areas	Strains	Heavy metals mg·mL^−1^				
		Pb	Cu	Zn	Cr	Cd
Tenfit mine	GT6, GT15, GT39	0.25	0.10	0.10	0.15	—
	GT14, GT41	0.10	0.10	0.10	0.10	—
	GT1	0.25	0.10	—	0.15	—
	GT2, GT38	0.25	0.10	—	0.10	—
	GT44	0.20	0.10	—	0.10	—
	GT12, GT13, GT3	0.10	0.10	—	0.10	—
	GT4, GT10	0.30	—	—	0.10	—
	GT40	0.25	—	—	0.10	—
	GT5	0.20	—	—	0.10	—
	GT7, GT8, GT9, GT11	0.10	—	—	0.10	—

Sidi Bouatman	SB20, SB21	0.30	0.10	0.10	0.10	—
	SB16, SB18, SB30, SB31	0.30	—	0.10	0.15	—
	SB19, SB22	0.30	—	—	0.15	—

Bir nhass	BN26	0.30	0.10	0.10	0.15	—
	BN2	0.55	—	—	0.15	—
	BN5, BN9, BN12, BN23, BN46, BN56, BN69, BN71, BN82	0.30	—	—	0.15	—
	BN3, BN4, BN13, BN70, BN72, BN73	0.30	—	—	0.10	—
	BN7, BN17, BN22, BN24, BN25, BN27, BN28, BN29, BN68	0.25	—	—	0.10	—
	BN47, BN48, BN57, BN58	0.20	—	—	0.10	—

(—): no tolerant strains.

**Table 3:** Growth percentages of actinobacteria isolated from mining areas.

Heavy metal	Mining areas	Number of actinobacteria isolated	Concentration of heavy metals			
			0.05	0.15	0.25	1
			mg·mL^−1^ ^a^	mg·mL^−1^ ^a^	mg·mL^−1^ ^a^	mg·mL^−1^ ^a^
Lead	Bir Nhass	30	100	100	56.67	—
	Sidi Bouatman	8	100	100	100	—
	Tenfit mine	21	100	52.38	9.52	—
	Total	**59**	**100**	**84.13**	**55.40**	**—**

Cadmium	Bir Nhass	30	—	—	—	—
	Sidi Bouatman	8	—	—	—	—
	Tenfit mine	21	—	—	—	—
	Total	**59**	**—**	**—**	**—**	**—**

Copper	Bir Nhass	30	3.33	—	—	—
	Sidi Bouatman	8	25.00	—	—	—
	Tenfit mine	21	61.90	—	—	—
	Total	**59**	**30.08**	**—**	**—**	**—**

Zinc	Bir Nhass	30	3.33	—	—	—
	Sidi Bouatman	8	75	—	—	—
	Tenfit mine	21	23.81	—	—	—
	Total	**59**	**34.05**	**—**	**—**	**—**

Chromium	Bir Nhass	30	100	—	—	—
	Sidi Bouatman	8	100	—	—	—
	Tenfit mine	21	100	—	—	—
	Total	**59**	**100**	**—**	**—**	**—**

^a^Results are expressed in percentage of strains grown on culture medium with metal per total tested actinobacteria from each mining residue.

(—): no tolerant strains.

**Table 4:** Biochemical and morphological characteristics of 27 metal accumulating isolates.

Characteristics	Strains																										
	BN3	BN4	BN24	BN68	BN72	BN13	BN17	BN22	BN25	BN48	BN7	BN73	GT1	GT2	SB30	GT15	GT6	GT39	BN82	BN2	BN9	BN12	BN69	BN71	SB22	BN23	SB31
ISP2	**++**	**+++**	**++**	**+++**	**+++**	**+++**	**+++**	**+++**	**+++**	**+++**	**+++**	**+++**	**+++**	**+++**	**+++**	**+++**	**+++**	**+++**	**+++**	**+++**	**+++**	**+++**	**+++**	**+++**	**+++**	**+++**	**+++**
ISP4	**+++**	**+++**	**++**	**+++**	**+++**	**+++**	**+++**	**+++**	**+++**	**+++**	**+++**	**+++**	**+++**	**+++**	**+++**	**+++**	**+++**	**+++**	**+++**	**+++**	**++**	**++**	**+++**	**+++**	**+++**	**+++**	**+++**
ISP5	**+++**	**+++**	**+++**	**+++**	**+++**	**+++**	**+++**	**+++**	**+++**	**+++**	**+++**	**+++**	**+++**	**+++**	**+++**	**+++**	**+++**	**+++**	**+++**	**+++**	**+++**	**+++**	**+++**	**+++**	**+++**	**+++**	**+++**
Arial spore mass	W	G	Ow	Ow	G	W	G	G	G	G	G	V	Pi	W	C	W	W	W	W	W	W	W	W	W	W	W	C
Colony reverse	B	T	Y	B	B	B	T	Gr	T	B	B	P	B	B	Lb	B	Lb	Lb	B	B	T	T	T	B	T	B	Lb
Soluble pigment	**−**	**−**	**−**	**−**	**−**	**−**	**−**	**−**	**−**	**−**	**−**	**−**	**−**	**−**	**−**	**−**	**−**	**−**	**−**	**−**	**−**	**−**	**−**	**−**	**−**	**−**	**−**
Melanin on tyrosine agar	**−**	**−**	**−**	**−**	**−**	**−**	**−**	**−**	**−**	**−**	**−**	**−**	**−**	**−**	**−**	**−**	**−**	**−**	**−**	**−**	**−**	**−**	**−**	**−**	**−**	**−**	**−**
C source utilization																											
Sucrose	**−**	**−**	**−**	**−**	**±**	**−**	**−**	**+**	**−**	**+++**	**−**	**+**	**+**	**+**	**−**	**++**	**+++**	**+++**	**−**	**−**	**+**	**−**	**+++**	**+++**	**−**	**−**	**−**
Fructose	**−**	**−**	**−**	**−**	**+**	**++**	**++++**	**−**	**−**	**++**	**++**	**+++**	**+++**	**+++**	**+++**	**+++**	**+++**	**+++**	**−**	**−**	**−**	**±**	**−**	**++**	**+**	**−**	**−**
Glucose	**+**	**+**	**+**	**+**	**+**	**+**	**+**	**+**	**+**	**+**	**+**	**+**	**+**	**+**	**+**	**+**	**+**	**+**	**+**	**+**	**+**	**+**	**+**	**+**	**+**	**+**	**+++**
Arabinose	**−**	**−**	**−**	**−**	**−**	**−**	**−**	**+++**	**−**	**+**	**+**	**−**	**+++**	**+++**	**−**	**+++**	**+++**	**+++**	**−**	**−**	**−**	**+**	**+**	**+**	**−**	**−**	**+**
Maltose	**−**	**−**	**−**	**−**	**−**	**+**	**+++**	**+++**	**−**	**+++**	**+++**	**++**	**+++**	**+++**	**−**	**+++**	**+++**	**+++**	**−**	**−**	**−**	**−**	**−**	**++**	**+**	**−**	**−**
Mannose	**−**	**−**	**++**	**−**	**+**	**−**	**−**	**+**	**−**	**−**	**−**	**+**	**++++**	**++++**	**−**	**+++**	**++**	**++**	**+**	**−**	**−**	**±**	**+**	**+++**	**−**	**−**	**−**
Mannitol	**−**	**−**	**++**	**−**	**++**	**+**	**++**	**+++**	**−**	**+++**	**−**	**++**	**+++**	**+++**	**+**	**++**	**+++**	**+++**	**−**	**−**	**−**	**±**	**+++**	**+++**	**−**	**−**	**−**
Galactose	**±**	**±**	**±**	**−**	**−**	**+**	**+++**	**±**	**−**	**+++**	**−**	**+++**	**++**	**++**	**+**	**+++**	**+++**	**+++**	**−**	**−**	**−**	**±**	**−**	**+++**	**−**	**−**	**+**
Inositol	**−**	**±**	**++**	**−**	**−**	**−**	**+**	**++**	**−**	**++**	**++**	**++**	**+++**	**+++**	**+**	**+++**	**+++**	**+++**	**−**	**−**	**−**	**+**	**+++**	**−**	**−**	**−**	**+**
Rhamnose	**−**	**−**	**−**	**−**	**−**	**−**	**−**	**−**	**−**	**−**	**−**	**−**	**−**	**−**	**−**	**+++**	**+**	**+**	**−**	**−**	**−**	**−**	**+**	**−**	**−**	**−**	**+**
Sorbitol	**−**	**−**	**−**	**−**	**−**	**+**	**−**	**−**	**−**	**−**	**−**	**++**	**+**	**+**	**−**	**+++**	**+++**	**+++**	**−**	**−**	**++**	**−**	**+**	**±**	**−**	**−**	**−**
Lactose	**−**	**−**	**−**	**−**	**−**	**−**	**+**	**−**	**−**	**+**	**+**	**+++**	**+++**	**+++**	**++**	**+++**	**+**	**−**	**−**	**−**	**++**	**+++**	**+++**	**+++**	**+**	**−**	**−**
Dextrin	**−**	**−**	**−**	**−**	**−**	**−**	**−**	**−**	**−**	**−**	**−**	**−**	**+++**	**+++**	**−**	**+**	**+**	**+**	**−**	**−**	**−**	**−**	**−**	**−**	**−**	**−**	**++**

“+” tested positive/utilized as substrate; “−” tested negative/not utilized as substrate; “**±**” low growth.

B: brown; C: colorless; G: gray; Gr: green; Ow: off white; Lb: light brown; P: purple; Pi: pink; T: tan; V: violet; W: white Y: yellow.

**Table 5:** Comparison of percent similarities between our 16S rRNA gene sequence and sequences present in the genomic database banks using NCBI BLAST.

Strains	Percentage of sequence identities (%)	Actinomycetes strains	Accession number
*Streptomyces* sp. BN2 *Streptomyces *sp. BN9 *Streptomyces *sp. BN12 *Streptomyces *sp. BN23 *Streptomyces *sp. BN71 *Streptomyces *sp. SB22	99	*Streptomyces *sp. strain NT307(K3) *Streptomyces albus *subsp.* albus *(NRRL B-2365) *Streptomyces flocculus* (NBRC 13041)	AJ002083.1 DQ026669.1 NR_041100.1

*Streptomyces *sp. BN7	99	*Streptomyces *sp. HB096 *Streptomyces diastaticus *subsp.* diastaticus *(NBRC 3714)	GU213492.1 NR041209.1

*Streptomyces *sp. BN13 *Streptomyces *sp. BN17	99	*Streptomyces gougerotii *(NBRC 13043) *Streptomyces *sp. HB096	NR112610.1 GU213492.1

*Streptomyces *sp. BN22 *Streptomyces *sp. BN25 *Streptomyces *sp. BN48	100	*Streptomyces *sp. HB096 *Streptomyces rutgersensis *(NBRC 12819)	GU213492.1 NR041077.1

*Streptomyces *sp. BN3 *Streptomyces *sp. BN24 *Streptomyces *sp. BN68	100	*Streptomyces* sp. (VTT E-062974) *Streptomyces fungicidicus *(YH04) *Streptomyces *sp. L116	EU430546.1 AY636155.1 EU410509.1

*Streptomyces *sp. BN4	99	*Streptomyces *sp. (VTT E-062974) *Streptomyces *sp. L116	EU430546.1 EU410509.1

*Streptomyces *sp. BN69	99	*Streptomyces albus *subsp.* albus *(NRRL B-2365) *Streptomyces gibsonii* (NBRC 15415)	DQ026669.1 NR041180.1

*Streptomyces *sp. BN72	99	*Streptomyces sampsonii *strain S151A *Streptomyces *sp. VTT E-062974	HQ439905.1 EU430546.1

*Streptomyces *sp. BN73	100	*Streptomyces rochei *strain A-1 *Streptomyces *sp. (NEAU-L11)	GQ392058.1 JF502572.1

*Streptomyces *sp. BN82	99	*Actinobacterium *BH0951 *Streptomyces aurantiogriseus *strain NRRL B-5416	GU265720.1 AY999773

*Streptomyces *sp. GT1 *Streptomyces *sp. GT2	99	*Streptomyces *sp. CS38 *Streptomyces kanamyceticus *(NRRL B-2535) *Streptomyces aureus *(NBRC 100912) *Streptomyces kanamyceticus *(NBRC 13414)	EF494232.1 NR043822.1 NR112608.1 NR112397.1

*Amycolatopsis *sp. GT6 *Amycolatopsis *sp. GT15 *Amycolatopsis *sp. GT39	99	*Amycolatopsis *sp. SA08 *Amycolatopsis decaplanina *(DSM 44594) *Amycolatopsis japonica *(MG 417-CF 17) *Amycolatopsis keratiniphila *subsp.* nogabecina *(DSM 44586) *Amycolatopsis orientalis *(IMSNU 20057)	GU294685.1 NR025562.1 NR025561.1 NR025562.1 NR042040.1

*Streptomyces *sp. SB30 *Streptomyces *sp. SB31	99	*Streptomyces pluricolorescens *(NAPOLSKAYA1, isolate 2) *Streptomyces parvus *(13647J) *Streptomyces praecox *(CGMCC 4.1782 clone 2)	FR837631.1 EU741140.1 JQ924403.1

**Table 6:** Heavy metal concentrations (mg·mL^−1^) available in mining residues [21].

	Goundafa	Sidi Bouatman	Bir Nhass
Zn	3.600	8.406	7.671
Pb	1.280	1.922	0.065
Cu	0.022	0.023	0.110
Cd	0.043	0.174	0.054
